# Medical Periodical Literature

**Published:** 1846-11

**Authors:** 


*Medical Periodical Literature.*—In the absence of other topics, suggesting themselves from their greater importance, it occurs to us, to indulge some lucubrations on the importance of *Medical Periodical Literature*, trusting that our readers will not attribute to us motives which relate to our own undertaking, farther than they, also, concern the general welfare of the profession.

The fact that periodical literature is of great value, as a means of developing and diffusing medical knowledge, and in various modes, advancing the great objects and interests of the medical profession, will not be questioned. But there is reason to believe that its advantages are not generally sufficiently appreciated by the medical profession. As evidence of this, we have only to refer to the difficulty with which Journals generally succeed in securing and maintaining existence—not to speak of a degree of success commensurate with the amount of labor and expense which they involve, We attribute this fact in part, to the present condition of the medical profession, arising in a great degree from the estimation in which it is held by the public. The absence of that stimulus to improvement which would spring from a better and more general appreciation of medical qualifications; the discouragement, and the temptation to indolence resulting from the reflection, that cultivated abilities, and learned acquirements, furnish no reliable security against neglect—these, and other circumstances tending to impoverish the profession, and depreciate the standard of attainment, affect the success of all means for promoting and disseminating medical knowledge. But the fact of which we are speaking is not attributable to these causes alone. The importance of sustaining periodical literature is not always duly appreciated, even by those who occupy positions which remove them beyond these considerations, and who are really disposed to do what they can for their own improvement, and for the advancement of the profession. If medical men of this class generally felt an interest in medical journals, sufficiently to supply contributions for their pages, and, at all events, not to fail of bestowing their patronage as subscribers, a new era would dawn on periodical literature, and its beneficial results would be incomparably greater than they now are.

It is through the instrumentality of medical periodicals only, that facts and ideas originating among the widely scattered members of the profession, can be collected, and combined for common availability. Our science is peculiar in the conditions of its prosecution. It differs, from most other departments of knowledge in relating to phenomena which can only be studied as they unexpectedly make their appearance. They cannot be pre-determined, and re-produced by experiment. They rarely occur twice under precisely the same circumstances. It is, in short, based upon the aggregated, cumulative experience of its numerous votaries. While all are not equally favored as regards opportunities for its cultivation, and while it affords ample scope for the exercise of transcendent talent, nevertheless, with ordinary abilities, and a limited range for observation, much may be contributed towards its progress, and each individual member of the profession, if he be disposed, can throw in his mite. How are these widely scattered elements and materials to be gathered, preserved, and rendered subservient to useful results, except by the agency of medical periodicals? It is plain that a large portion will never reach the medical public in books unless by passing first through this channel. They cannot circulate extensively by simply becoming topics of conversation. Physicians even of the same district, from various causes, seldom assemble for colloquial intercourse: and if it were otherwise, it would be impossible in this way alone, to accomplish the end to any considerable extent. The office of a journal is to afford facilities for that intercommunion among the members of the profession, which is so desirable and important. One of its most valuable functions is to furnish a ready, accessible mode of transportation of supplies from every quarter, to be concentrated, and made available for the medical public. The parallel holds good between the operation of these highways of knowledge, and the advantages of railroads and canals in commercial affairs. The latter serve to develop resources in widely separated localities, enlarge the sphere of competition, stimulate and quicken individual industry and enterprise, and promote, in various ways, public and private wealth. Analogous results in the dominion of science and mind, are derived through the agency of periodical literature.

These remarks apply especially to practical medicine. A vast amount of valuable matter, both of thought and fact, exists among the active practical members of the profession, which is confined to individua's, or, at most, extends to the circle of immediate friends. Practical medicine as it exists in books is by no means a fair representation of practical medicine as exemplified in the actual practice of the profession. The reason of this is, that the fruits of individual reflection and experience often remain private property, not being contributed to the common stock of knowledge. Individuals are too apt to retain what they have acquired, which by themselves, at least, is deemed valuable truth, as if it were like worldly wealth, holding it for their own exclusive benefit. But in so doing they are unjust to themselves as well as to others. By giving it to the public its value to them would be unimpaired, while all would participate in its benefits; and, in return, by becoming joint recipients of the contributions of others, their own are repaid an hundred fold.

These few remarks on a topic which admits of wide amplification, we make merely as suggestives for the reflections of our readers. We have only referred to its relations in a single aspect. This aspect is one of the most prominent, but there are others in which its importance is equally obvious, and in which it is too little regarded. Upon these we may perhaps comment hereafter.

Regarding the cultivation and support of periodical literature as one of the most efficient means of advancing medicine, as a science and as a profession, to a higher position than, in either point of view, it now occupies, we look upon the establishment of Medical Journals at favorable points of location, as a measure of very great importance. So far from conflicting with each other, and mutually suffering by competition, if they tend (as they doubtless will) to increase the general estimation of their usefulness, they will assist and strengthen each other. The advantages of proximity, from the greater facilities offered to contributors, the incitements to scientific investigations, the development of an honorable spirit of emulation, and other local considerations, render their multiplication at different points highly desirable. The sectional support which they receive, instead of deducting from that bestowed upon others, will be so much additional patronage and encouragement of periodical literature, which otherwise would be entirely lost. While the influence of different Journals in their immediate neighborhood will serve to increase the amount of material for those which have a general or national character, they will, also, by stimulating the desire for improvement, and by contributing to the standard of medical attainments, tend to enhance the general appreciation of the value of the latter, and thus directly and efficiently promote their interests.

